# DALI: Vitamin D and lifestyle intervention for gestational diabetes mellitus (GDM) prevention: an European multicentre, randomised trial – study protocol

**DOI:** 10.1186/1471-2393-13-142

**Published:** 2013-07-05

**Authors:** Judith GM Jelsma, Mireille NM van Poppel, Sander Galjaard, Gernot Desoye, Rosa Corcoy, Roland Devlieger, Andre van Assche, Dirk Timmerman, Goele Jans, Jurgen Harreiter, Alexandra Kautzky-Willer, Peter Damm, Elisabeth R Mathiesen, Dorte M Jensen, Liselotte Andersen, Fidelma Dunne, Annunziata Lapolla, Graziano Di Cianni, Alessandra Bertolotto, Ewa Wender-Oegowska, Agnieszka Zawiejska, Kinga Blumska, David Hill, Pablo Rebollo, Frank J Snoek, David Simmons

**Affiliations:** 1 Department of Public and Occupational Health, EMGO+−Institute for Health and Care Research, VU University Medical Centre, Van der Boechorststraat 7, 1081BT Amsterdam, the Netherlands; 2 KU Leuven Department of Development and Regeneration: Pregnancy, Fetus and Neonate, Gynaecology and Obstetrics, University Hospitals Leuven, Leuven, Belgium; 3 Department of Obstetrics and Gynecology, Medizinische Universitaet Graz, Graz, Austria; 4 Institut de Recerca de l’Hospital de la Santa Creu i Sant Pau, Barcelona, Spain; 5 CIBER Bioengineering, Biomaterials and Nanotechnology, Instituto de Salud Carlos III, Madrid, Spain; 6 Medical University of Vienna, Vienna City, Austria; 7 Center for Pregnant Women with Diabetes, Rigshospitalet, University of Copenhagen, Copenhagen, Denmark; 8 Odense University Hospital, Odense, Denmark; 9 National University of Ireland, Galway, Ireland; 10 Universita Degli Studi di Padova, Padua, Italy; 11 Università di Pisa, Pisa, Italy; 12 Uniwersytet Medyczny im Karola Marcinkowskiego W Poznaniu, Poznan, Poland; 13 6 Recherche en Santé Lawson SA, Bronschhofen, Switzerland; 14 BAP Health Outcomes Research SL, Oviedo, Spain; 15 Department of Medical Psychology, EMGO+−Institute for Health and Care Research, VU University Medical Centre, Amsterdam, the Netherlands; 16 Institute of Metabolic Science, Addenbrookes Hospital, Cambridge, England

**Keywords:** Gestational diabetes mellitus, Pregnancy, Lifestyle intervention, Randomised controlled trial, Healthy eating, Physical activity, Overweight, Motivational interviewing, Prevention, Vitamin D

## Abstract

**Background:**

Gestational diabetes mellitus (GDM) is an increasing problem world-wide. Lifestyle interventions and/or vitamin D supplementation might help prevent GDM in some women.

**Methods/design:**

Pregnant women at risk of GDM (BMI≥29 (kg/m^2^)) from 9 European countries will be invited to participate and consent obtained before 19+6 weeks of gestation. After giving informed consent, women without GDM will be included (based on IADPSG criteria: fasting glucose<5.1mmol; 1 hour glucose <10.0 mmol; 2 hour glucose <8.5 mmol) and randomized to one of the 8 intervention arms using a 2×(2×2) factorial design: (1) healthy eating (HE), 2) physical activity (PA), 3) HE+PA, 4) control, 5) HE+PA+vitamin D, 6) HE+PA+placebo, 7) vitamin D alone, 8) placebo alone), pre-stratified for each site. In total, 880 women will be included with 110 women allocated to each arm. Between entry and 35 weeks of gestation, women allocated to a lifestyle intervention will receive 5 face-to-face, and 4 telephone coaching sessions, based on the principles of motivational interviewing. The lifestyle intervention includes a discussion about the risks of GDM, a weight gain target <5kg and either 7 healthy eating ‘messages’ and/or 5 physical activity ‘messages’ depending on randomization. Fidelity is monitored by the use of a personal digital assistance (PDA) system. Participants randomized to the vitamin D intervention receive either 1600 IU vitamin D or placebo for daily intake until delivery. Data is collected at baseline measurement, at 24–28 weeks, 35–37 weeks of gestation and after delivery. Primary outcome measures are gestational weight gain, fasting glucose and insulin sensitivity, with a range of obstetric secondary outcome measures including birth weight.

**Discussion:**

DALI is a unique Europe-wide randomised controlled trial, which will gain insight into preventive measures against the development of GDM in overweight and obese women.

**Trial registration:**

ISRCTN70595832

## Background

Europe is facing an unprecedented threat from Type 2 diabetes (T2D) with associated human suffering and an economic burden of enormous and rapidly growing proportions [1]. While T2D is traditionally associated with a sedentary lifestyle and an unhealthy diet, the currently observed growth in developed countries is greater than expected from lifestyle deficiencies alone. Evidence is accumulating that Gestational Diabetes Mellitus (GDM) may be a more important contributor to these epidemics than previously recognised [2,3]. Firstly, women with past GDM comprise up to 31% of parous women with T2D [4]. Secondly, intrauterine exposure to hyperglycaemia through GDM, predisposes the off-spring to diabetes and obesity: so called “fuel mediated teratogenesis” [5]. If GDM is acting as the “accumulator” contributing to the T2D epidemic, strategies to arrest this inter-generational transmission are urgently needed.

GDM is defined as ‘carbohydrate intolerance resulting in hyperglycaemia of variable severity with onset or first recognition during pregnancy’ [6]. GDM is characterised by pancreatic beta cell function that is insufficient to meet the body’s insulin needs, usually in association with the increasing insulin resistance of pregnancy. There is no common unique pathogenic complication of diabetic pregnancy and a continuous relationship exists between maternal glycaemia and perinatal outcomes [7].

Like T2D in general, the prevalence of GDM in Europe is reported to vary considerably, in some populations GDM occurs already in up to 20% of all pregnancies. However, there is no generally accepted and uniform European agreement on screening approaches and diagnostic standards, making pan-European surveys of GDM currently very difficult [8].

The pathophysiological processes associated with GDM, particularly those relating to insulin secretory capacity and underlying insulin resistance are also contributors to the development of T2D. Strategies for preventing T2D could therefore also be useful for GDM prevention. Currently no strong evidence exists regarding the best intervention for prevention of GDM [9,10], although a low glycaemic index diet, healthy diet according to recommendations for the general population, or an exercise program could be beneficial. In addition lifestyle studies conducted specifically with overweight or obese pregnant women, potentially involving weight gain restriction, may reduce the prevalence of GDM and restrict gestational weight gain, although the quality of the published studies thus far is mainly poor [11]. Well designed randomised trials, with standardised behavioural interventions are needed [12].

Beyond lifestyle interventions, Vitamin D supplementation (serum 25-hydroxyvitamin D (25OHD)) has also been proposed as an approach that could prevent T2D [13]. In pregnancy, insulin sensitivity is reduced. Normally, improved beta cell function and proliferation meet the increased secretory demands in pregnancy, but when this fails, GDM will occur. In pregnant women, an inverse correlation was found between vitamin D and insulin resistance, measured using Homeostasis Assessment (HOMA), suggesting that deficiency may contribute to insulin resistance [14]. In women with GDM, administration of vitamin D led to a decrease in fasting glucose levels [15]. Therefore, vitamin D is postulated to contribute to insulin sensitivity and beta cell function, and deficiency may contribute to impaired glucose tolerance (IGT) during pregnancy. Suggested mechanisms are varied and in addition to those mediated through calcium and parathyroid hormone, include effects on cytokine release and innate and adaptive immunity [16].

This study will focus on prevention of GDM in the antenatal period and is designed to collate evidence about the epidemiology of GDM in Europe, to promote pan-European standards and measures for GDM and to identify suitable preventive measures against GDM.

## Methods/design

### Overall study design

This is a multicentre, randomized controlled trial using a 2×(2×2) factorial design across nine European countries. Pregnant women attending a participating antenatal clinic or hospital in one of these countries will be approached and asked to take part in the study. Baseline assessment will occur before 20 weeks gestation, immediately followed by randomization into one of the eight, pre-stratified intervention groups. Follow up measurements will occur at 24–28 weeks, 35–37 weeks and birth. This trial is funded by the European Union 7th framework (FP7/ 2007–2013) under Grant Agreement no. 242187, further local funding is received in some of the individual centres. All study procedures have been pilot tested and modified as necessary for the main study. The study was approved by the relevant ethical committees before the start of DALI (NRES Committee East of England – Norfolk: 11/EE/0221; Medical University Poznan: 1165/12; UZ KU Leuven: ML7625; VUmc Amsterdam: 2012/400; Hospital De La Santa Creu i Sant Pau Barcelona 13/006 (OBS); Medical University Vienna: 2022/2012 – 1369/2013; Region Hovedstaden Copenhagen: H-4-2013-005; Province of Padua: 4201 × 11; Galway University Hospitals: 7/12).

### Participants

Pregnant women with a pre-pregnancy body mass index (BMI) ≥ 29 kg/m^2^ are eligible for inclusion [17]. Where pre-pregnancy weight is not known the most earliest antenatal weight will be used. Further inclusion criteria are: before 19+6days of gestation, singleton pregnancy and aged ≥ 18 years.

Women will be excluded from the study if they: are diagnosed with GDM on oral glucose tolerance testing, before randomization, using IADPSG criteria defined as fasting venous plasma glucose ≥ 5.1 mmol/l and/or 1 hour glucose ≥10 mmol/l and/or 2 hour glucose ≥ 8.5 mmol/l at baseline measurement [18]; have pre-existing diabetes; are not able to walk at least 100 meter safely; require complex diets; have chronic medical conditions (e.g. valvular heart disease); have significant psychiatric disease; are unable to speak major language of the country of recruitment fluently or are unable to converse with the lifestyle coach in another language for which translated materials exist. For the vitamin D arm, two additional exclusion criteria apply: have current or past abnormal calcium metabolism, e.g. hypo/hyperparathyroidism, nephrolithiasis, hypercalciuria; have hypercalciuria (>0.6 mmol/mmol creatinine in spot morning urine) or hypercalcaemia (>10.6 mg/dl | 2.65 mmol/l) detected at baseline measurement.

Women who have developed GDM at baseline are informed that they can not participate any further in DALI and are recommended to contact their health care provider regarding their GDM. These women are asked to consent to have the information regarding their pregnancy outcomes collected. As the IADPSG criteria are not used to diagnose GDM at all sites, each site has a protocol on how to link women with appropriate services in their locality.

#### Sample size and power calculation

The primary outcomes of the study are gestational weight gain, fasting glucose and insulin sensitivity in late pregnancy. The numbers needed in each arm (80% power, 5% significance, two tailed alpha) were calculated assuming a 20% drop out. To detect a weight gain difference of 4 kg (mean of 11 kg and standard deviation (SD) of 6.5 kg) we would need 80 women in each arm. To measure a fasting glucose difference of 0.3 mmol/l (mean of 5.0 mmol/l and a SD of 0.5 mmol/l) we would need 85 women in each arm. To find a difference 0.44 for the HOMA-IR (mean of 2.2 and a SD of 0.8) we would need 101 women in each arm. Lesser numbers are required when using the factorial design, combining cells for comparisons. The trial will be conducted in nine European countries (ten study centres) and in each centre 88 women will be recruited (total n = 880) before 20 weeks of pregnancy and randomly assigned to one of the eight arms pre-stratified for centre and 2x2 trial (lifestyle 2x2 or vitamin D/placebo 2x2).

### Recruitment

Women will be recruited in the following nine European countries: United Kingdom, Ireland, Netherlands, Belgium, Poland, Italy, Spain, Austria, Denmark (2 study centres), from a diverse range of socio-economic and ethnic backgrounds. Participating hospitals, surrounding midwives, obstetricians and general practices are involved in recruitment. Each country will have its own tactics to optimize recruitment (e.g. leaflets, flyers, posters outlining the intervention). An information sheet will be given to all eligible participants, which includes all aspects of the study. Women will have the opportunity to consider participation and to ask questions to the researcher. It is stressed that women can terminate participation at any time within the study without affecting her usual care. The privacy of the participant is protected, and all data will be protected and processed anonymously. Written consent will be obtained prior to the first baseline measurement. Women are requested to give consent for 1) participation in the study, including gathering data, blood sampling, associated measurements and extraction of relevant data from medical records, 2) storage of blood and placenta tissue in a biobank and 3) external examination of their baby at birth. Depending on the site, costs involving study visits, parking or travel expenses will be reimbursed and in some places small gifts for attending the measurement visits will be given. The inclusion period is planned from February 2013 until the end of December 2013, based on recruitment of 2–3 patients a week at each centre. At each site a screening log will contain all patients screened for the study and the reasons why they were not randomised, if this is the case, to allow the consort diagram to be completed.

### Randomisation

**Figure 1:**
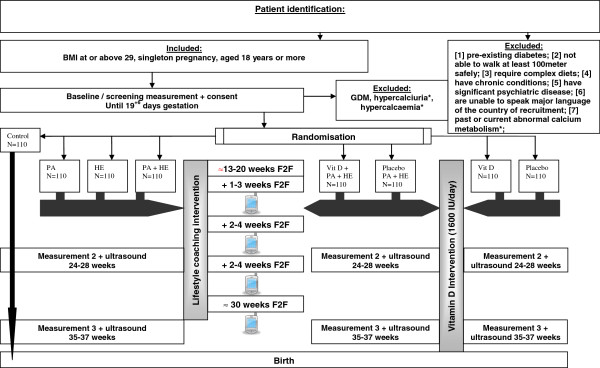
**Trial schedule.** * vitamin D arm; PA= Physical Activity; HE= Healthy Eating; Vit D = Vitamin D; GDM= Gestational Diabetes Mellitus; F2F = Face to Face; picture phone = telephone call.

Women who are eligible will be randomly allocated to one of the eight intervention arms (as is shown in Figure 1): 1) healthy eating (HE), 2) physical activity (PA), 3) HE+PA, 4) HE+PA+vitamin D, 5) HE+PA+placebo, 6) vitamin D alone, 7) placebo alone, 8) control. A computerized random number generator draws up an allocation schedule pre-stratified for intervention centre and 2x2 trial. The DALI trial coordinator will prepare sealed opaque envelopes containing the intervention arm to which each participant is allocated. The outcome of the allocation will be reported by the lifestyle coach to the participant, before the start of the intervention. Those involved with measurements are kept blinded of the intervention.

### Procedure

Clinical assessments will be made by a member of the research team at four time points: before 20 weeks (Screening/baseline), 24–28 weeks (visit 2), 35–37 weeks (visit 3) gestation and after delivery. The lifestyle intervention will be delivered by a lifestyle coach (Figure 1). Vitamin D will be taken daily from randomisation until delivery.

Should GDM develop after baseline, women will be managed according to local standard practice. All treatments will be recorded and the DALI intervention will be discontinued. Measurement procedures, however, will continue, although at 35–37 weeks instead of an oral glucose tolerance test (OGTT) only fasting blood will be collected.

### Lifestyle interventions

After randomization, and after receiving all baseline measurement results, the individual sessions with a lifestyle coach will be scheduled for each participant. The coaching is targeted at healthy eating and/or physical activity according to a defined schedule. It builds on principles of patient empowerment and cognitive behavioural techniques, inspired by Motivational Interviewing (MI). Women will be assigned to the same personal coach during the intervention. The overarching intervention framework was derived from a previous lifestyle trial aimed at diabetes prevention in (non-pregnant) adults [19].

In the programme, one-to-one contact will be offered, along with telephone booster calls. The same amount of time will be offered to every participant during the trial. The intervention will be provided in five sessions of approximately 30–45 minutes duration, and in four optional telephone calls of up to 20 minutes that occur between the one-to-one sessions (see Figure 1). The one-to-one sessions can take place either in the home of the participants or in the hospital/midwife practice/general practice, depending on local arrangements. The timing of these contacts and the time between these contacts is dependent on the preference of the participant and the availability of the lifestyle coach, in order to deliver the intervention with the most appropriate timing and to optimize chances of the uptake of behaviour change. However it is stressed that at least 4 face-to-face coaching sessions should occur before the second measurement and that the intervention should be finished before 35 weeks of gestation.

To facilitate the coaches and promote treatment integrity, each coach will have a desk-file outlining the intervention and options in detail and will use a Personal Digital Assistant (PDA) to provide a framework for the visit and to help guide the coach to deliver the intervention. The PDA will also be used to record the intervention results / discussion at each step. Details of the session will be entered into the PDA programme as the session progresses or directly at the end of the session in order to focus all attention on the participant. All information will be synchronised to the server of the trial coordination team at the end of each session.

To prepare consultations, answers from the baseline questionnaire on the ‘readiness to change scores’ for all the physical activity and healthy eating messages will be loaded on the PDA and can be used in helping to tailor the intervention, e.g. explain risk factors and identify barriers to change. Participants will choose one or more key items (agenda setting) as their main area of concern: risk factors of GDM, weight management, healthy eating and/or physical activity. The last two can only be chosen once subjects are allocated to these intervention groups.

Once a message has been selected, the first phase of behaviour change is centred on intention formation (motivation). Current behaviour will be assessed and motivation for change will be established. To assess the participants’ readiness to change, coaches can use a ruler for motivation, importance and confidence, prompting the question: “How motivated are you right now to change this specific behaviour on a scale from 1–10, where 1 is not at all motivated, and 10 extremely motivated”. Followed by questions to elicit change talk as “what is the reason you rated yourself at a ‘(for example 4)’ instead of a 1?” and “what do you need to change this 4 to a 6 (number rated as sufficient)?”. These scales are a useful tool for the coach as well as for the participant to gain insight in why change is needed (importance), what is driving the women’s desire to change (motivation) and to increase a person’s own beliefs of own ability to actually make the desired change (confidence). Since an individual’s readiness to change is variable and dynamic, these scales can be used in follow up consultations as well to gain insight in the development of the women’s changes she already experienced and serve as a feedback tool.

To help women resolve feelings of ambivalence and promote intrinsic motivation, perceived disadvantages and advantages of lifestyle change are explored using a decisional balance sheet. The primary focus is on reducing the disadvantages of the current behaviour and promoting the advantage of the preferred behaviour. The status quo will be acknowledged, while ‘change talk’ will be rewarded with positive reinforcement.

If women score low for the importance scale of changing their behaviour, education on risk and benefits might enhance their motivation for change. The communicated risk factors of developing GDM [20] consist of unchangeable (inherent) risk factors like: previous GDM, family history of diabetes, polycystic ovarian syndrome (PCOS), ethnic background as well as modifiable risk factors like: weight, unhealthy nutrition, insufficient physical activity and excessive sedentary behaviour. The primary focus of the intervention will be on the modifiable factors. The risk communication will serve as an introduction and will build motivation to change their weight, physical activity and nutrition behaviour.

By offering social support, encouraging women to say what could work, talking about past successes and achievements, speculating and exploring extremes, confidence for behaviour change will be enhanced.

Finally, when the woman is ready to make the change, this will be translated into personal goal setting and action planning. Action cards will be used as a tool to help women formulate a specific and realistic goal. Involving questions to alter the chances of success: “What is your action plan?, When will you start?, How will you make this change? What if it did not turn out the way you wanted, what will be your back-up plan?”.

Following realistic goal setting (in subsequent sessions), the women are encouraged and supported in their efforts to eat healthily and/or to be sufficiently physically active. During this action phase, behavioural strategies will be provided along with mobilising social support and identifying and overcoming obstacles where possible.

Participants allocated to one of the five groups with a lifestyle intervention receive a toolkit with useful materials to help them change their behaviour. The content of the toolkit is dependent on the group they are allocated to. All coached participants receive a participant manual, with general information about (risk of developing) GDM, which is explained above, and weight management.

The Institute of Medicine (IOM) has advised on a minimum, mean and maximum weight gain for each pre-pregnancy BMI-group until 40 weeks of gestational age. For the group ≥ BMI 29 it is advised to gain between 5 to 9 kilogram [21]. While the purpose of the DALI trial is to minimize gestational weight gain, women are advised to gain a maximum of 5 kilogram. If before the start of the intervention, they have already gained ≥ 5 kilogram the advice is to maintain this weight throughout the remaining pregnancy. The goal of the intervention is to make the women aware of the fact that they are able to influence their weight (gain) even during pregnancy. Goal setting to achieve optimal weight gain during pregnancy appears to be helpful [22]. Therefore, if women find it helpful to keep track of their weight, they have the possibility to measure their weight every session with the lifestyle coach.

Where a lifestyle choice is already in place, fortification discussions will occur. All messages will be reviewed as an option (although they may be promptly discarded). Some of the messages overlap, and so some actions will therefore address more than one message.

#### Physical activity (PA) intervention

According to the American College of Obstetrics and Gynaecology (ACOG) guidelines [23], pregnant women are recommended to be moderately physically active for at least 30 minutes per day (building up to 60 minutes if possible) on at least 5 days of the week (preferably 7).

Each participant will be advised by attractive messages to:

1. “Be active every day”: Incorporate light and moderate PA as much as possible into their daily life (e.g. by parking further away from destination or undertake special activities for pregnant women).

2. “Sit less”: Reduce sedentary time.

3. “Build your strength”: Incorporate upper and/or lower limb resistance exercise as PA.

4. “Take more steps”: To increase the number of steps taken per day.

5. “Be more active at weekends”: To be more active during the weekends.

An action plan for increasing PA levels will be made during the first session, and evaluated in subsequent sessions. The participants receive a manual with additional information about PA, including an adapted F.I.T.T. model (frequency, intensity, time, type) based on ACOG guidelines [23] and information about the above mentioned PA advices. To help the women increase their PA they will receive pedometers (Yamax Digiwalker SW-200, Tokyo, Japan) and flexible elastic dynabands (Thera-Band, Akron, USA), respectively to provide feedback about their behaviour and progress and to help them perform at home upper and/or lower limb resistance exercises. For these exercises, an additional (training) video has been developed, containing an 8 min workout with the dynaband and showing specific exercises one by one. The exercises can be found in the manual as well. Included in the manual is also a list with helpful places where pregnant women can go for PA classes, e.g. pregnancy swimming, pregnancy fitness classes and pregnancy yoga. Activities such as swimming, walking and cycling are activities that the participants should be able to undertake during the course of their pregnancy. As pregnancy progresses through the third trimester, PA may decrease and this will be managed sympathetically by providing alternatives e.g. upper limb exercises whilst sitting.

#### Healthy eating (HE) intervention

The following dietary objectives will be set for each participant to achieve or to maintain:

1. “Replace sugary drinks”: To reduce intake of sugary drinks (e.g. replace with water).

2. “Eat more non-starchy vegetables”: To eat more non-starchy vegetables.

3. “Increase fibre consumption”: To choose high-fibre, over low fibre products (≥5 g fibre/100 g).

4. “Watch portion size”: To be conscious about the amount of food eaten each meal.

5. “Eat protein”: To increase intake of proteins (e.g. meat, fish, beans).

6. “Reduce fat intake”: To reduce fat intake (e.g. snack, fast food, fried foods).

7. “Eat less carbohydrates”: To reduce intake of carbohydrates (e.g. potatoes, pasta, rice, snacks, candy).

An action plan for improving dietary behaviour will be made during the first session, and evaluated in subsequent sessions. Participants have the opportunity to discuss their food diary, which was filled out as a measurement tool in order to minimize time-consuming unnecessary intervention modules, with their coach. The participants receive the participant manual with additional information about healthy eating, including a section on how to read a food label, an adapted food pyramid (which is concurrent with the dietary objectives) and detailed information about the above-mentioned dietary topics.

##### Lifestyle coaches

A lifestyle coach will carry out the one-to-one coaching and the telephone booster sessions. The appointed lifestyle coaches have been selected for their natural ability to be emphatic, and most had a background in either behavioural change, healthy eating and/or physical activity. They will receive a special training program containing MI techniques [24] to help women overcome their ambivalence or barriers that keep them from making the desired lifestyle changes. The coaches receive the following tools: 1) a coach- and PDA manual in English, with detailed information about DALI, MI, a flow of a (‘perfect’) conversation containing helpful questions and background information and an explanation of the use of the PDA; 2) presentations in English about GDM; the DALI intervention and messages; MI; Goal setting/ planning; 3) a basic two to three day training course in MI from a professional local MI trainer in the local language of the country; 4) feedback on at least one actual conversation with a real person by a MI trainer preferably on the Motivational Interviewing Treatment Integrity (MITI 3.1.1) [25]. In the end all lifestyle coaches should be able to ‘roll with resistance‘, express empathy, develop discrepancy, support self-efficacy, elicit change talk, make complex reflections and ask open questions.

The coaching sessions will be supervised and evaluated by a local MI trainer to optimise coaching quality and to minimize variation between coaches. Each counselling session will be recorded to understand the interaction between the coach and participant, with the participant’s permission. The content, together with general characteristics, will be summarized in a report that is then filed in the database for future thematic analysis. In the pilot study lifestyle coaches have become acquainted with the protocol and the target group. Each coach has to confirm they have read the DALI study materials, provide written descriptions of some aspects of DALI, and attend an observed ‘standardisation session’ where their interaction with a mock participant is videoed. A standardisation meeting will take place at the beginning of the trial and a learning and sharing community page on *facebook* will provide an online place to post and discuss DALI related issues among lifestyle coaches in order to stimulate improvement of skills.

### Vitamin D intervention

No consensus on the definition of vitamin D (25-hydroxyvitamin D (25OHD)) sufficiency outside pregnancy exists, and cut-off levels of 50 nmol/L [26] and 75 nmol/L [27] have been proposed. There is no indication that the definition should be different in pregnancy, and in other pregnancy studies the same cut-offs for deficiency and insufficiency have been used as for non pregnant adults [28]. Thus, IOM recommendation is a minimum of 600 IU/day [29], National Institute for Health and Clinical Excellence (NICE) recommends a minimum of 400 IU/day [30] with the upper tolerable intake set at 4000 IU/day [29]. Normally obstetricians and midwives provide usual care including a prescription of multivitamin pills containing 400–600 IU vitamin D/day.

In a recent study no negative consequences occurred in pregnant women with 4000 IU vitamin D/day. It further showed that the desired plasma levels of 80 nmol/L were reached with 2000 IU/day and that a higher dose of 4000 IU/day did not increase plasma levels of vitamin D much more compared to 2000 IU/day [31].

Taking into account that most women use multivitamins during pregnancy, containing on average 400 IU vitamin D, we chose to use a dosage of 1600 IU/day as the intervention dosage in our trial, in tablet form, Devaron®, produced by Vemedia (Diemen, Netherlands) (RVG 09766). Each tablet contains 400 IU, and participants are asked to take 4 tablets/day until delivery. Devaron is prepacked in bottles containing 90 tablets each.

We aim at achieving a vitamin D concentration >=50 nmol/L at term in the majority of obese pregnant women receiving supplementation. Taking into account a non-confirmed publication of a U-shaped relationship between maternal vitamin D and offspring IgE [32], an upper cut-off 135 nmol/l (ideally not to exceed) will be considered.

### Placebo intervention

Placebo tablets, identical to the Devaron tablets in appearance, will be produced in bulk by Vemedia especially for the DALI trial. Vemedia will provide the results of the batch analysis of the placebo’s after producing those. An IMPD for the placebo will be produced by Apotheek Haagse Ziekenhuizen (AHZ). Packaging of the placebo will be done by AHZ in The Hague, in identical bottles with identical labels as the Devaron bottles. The women will be asked to take 4 tablets daily.

### Control group

The control group will receive the same recruitment process and study assessments as the other participants but they will not receive any lifestyle intervention or vitamin D/placebo administration. They will receive usual care from their midwife or obstetrician during pregnancy.

### Data collection

Data will be collected from participants at four time points (see Table 1): baseline/screening measurement (<20 weeks), at 24–28 weeks, at 35–37 weeks and after delivery. These visits are scheduled mostly together with usual care visits, to minimize time consuming study visits. At these time points (except at delivery) an OGTT is performed. In between blood collections, participants will be asked to complete a questionnaire and anthropometric measurements will be performed as well. The women will be asked to wear an accelerometer for 3 days including a weekend/holiday day and write down their nutritional intake in a food diary for that period. These data can be sent back with a reply-paid envelope which is provided to them. At first trimester, 24–28 weeks and 35–37 weeks, data of obstetrical ultrasound examinations will be collected, depending on the study site whether these check-ups are usual care visits or additional to the study.

**Table 1:** Sampling and measurements

	**Method or sample used**	**Baseline**	**24**-**28wk**	**35**-**37wk**	**birth**	**<48 hours postnatal**
**Physical examinations maternal**						
Weight	Scale (twice)	x	x	x		
Height	Stadiometer (twice)	x				
Waist circumference	Tape (twice)	x				
Neck circumference	Tape (once)	x	x	x		
Skin folds (fat percentage)	Calliper (twice)	x		x		
Blood pressure (systolic, diastolic) and heart rate	Twice	x	x	x		
**Physical examinations fetus**/**baby**						
Fetal ultrasound ^a^		x	x	x		
APGAR (1min; 5min)					x	
Neonatal measurements (birth weight, length, head circumference, neonatal body composition (skin fold thickness, circumference of extremities, abdominal circumference)	Calliper (twice) and tape					x
Perinatal and obstetric outcome: maternal and neonatal outcome ^f^ and complications					x	x
**Laboratory assessment**						
Fasting plasma glucose, Hb_A1c_, insulin ^b^	Fasting venous sample	x	x	x		x^g^
Leptin, vitamin A, vitamin D, carotenoids, 3ß-OH-Butyrate	Fasting venous sample	x	x	x		x^g^
Lipids (triglycerides, free fatty acids, total cholesterol, HDL cholesterol and LDL cholesterol)	Fasting venous sample	x	x	x		x^g^
(30), 60, (90), 120 min plasma glucose and serum insulin^b^	venous sample	x	x^c^	x^c^		
Serum calcium and albumin	Venous sample	x^d^	x^d^	x^d^		
Urinary calcium/creatinine ratio	Morning spot urine	x^d^	x^d^	x^d^		
Cord blood sample	Arterial and venous sample				x	
Placenta tissue	Tissue sample				x	
**Behaviour**						
Accelerometer 3 day	Actigraph	x	x	x		
PPAQ	Questionnaire	x	x	x		
Food diary 3 day	Food diary	x	x	x		
12 item food frequency list	Questionnaire	x	x	x		
Smoking	Questionnaire	x		x		
Alcohol	Questionnaire	x				
Sleeping pattern	Questionnaire	x	x	x		
Attitude, knowledge, social support, self-efficacy, risk perception and stages of change in PA and diet	Questionnaire	x	x	x		
Barriers and facilitators to change PA and HE	Questionnaire			x		
Access to PA venues and healthy food	Questionnaire	x	x	x		
**Vitamin D**						
Travel to warmer climate in winter/spring, sun beds	Questionnaire	x	x	x		
Pill count	Research nurse		x	x	x	
**Psychosocial**						
Health related Quality of life (EQ-5D)	Questionnaire	x	x	x	x	
WHO Five Well Being index	Questionnaire	x	x	x		
Cambridge Worry Scale	Questionnaire	x	x	x		
**Other**						
Household composition, marital status, ethnicity, education, employment, occupation, shift work, parity, age	Questionnaire	x				
Pre existing condition: family history DM, impaired glucose tolerance, hypertension, polycystic ovarian syndrome	Questionnaire	x				
Past/current obstetric history	Questionnaire	x				
Drugs/vitamins/medication	Questionnaire	x	x	x	x	
Adverse events and safety questions	Questionnaire		x	x	x^e^	
Questions about the biological father	Questionnaire	x				
**Evaluation**						
Dose, reach, fidelity, satisfaction	Questionnaire			x		
Costs^f^, distance, visits	Questionnaire	x	x	x		

^a^ Data collected from routine and expert scans that occur during the time points.

^b^ Blood samples will be collected in the fasting state and at [30, 60, 90 and 120 minutes] or [60, and 120 minutes] depending on site following ingestion of the glucose drink (75 g D-glucose in 250 ml water).

^c^ If GDM is diagnosed by IADPSG criteria, subsequent visits only test the fasting measurements.

^d^ obtained locally only for the vitamin D arm.

^e^ at delivery only registration of adverse events.

^f^ data registration for a longer period post natal.

^g^ collection of samples in a non fasting state.

*PPAQ* pregnancy physical activity questionnaire, *PA* physical activity, *HE* healthy eating, *WHO* world health organisation.

At birth maternal blood is collected shortly before the birth process (or a day after delivery, to minimize disrupting blood levels caused by the delivery itself). Placenta tissue and cord blood is collected and processed within 30 minutes after birth. Neonatal measurements will be performed within 48 hours after birth. The women will be asked to complete a questionnaire and data from the medical records will be requested regarding the delivery.

#### Blinding

Participants cannot be blinded for the intervention, but are asked not to reveal information about their intervention treatment to the measurement team. Across countries different health professionals perform the measurements. At the beginning of the trial and within 6 months all the health professionals are trained in a standardisation meeting to perform these measurements.

#### Physical examination

##### Maternal

Height will be measured only at the baseline measurement with a stadiometer (SECA 206, SECA, Birmingham, UK), with an accuracy to the nearest centimeter, the average value of two measurements will be used. Pre-pregnancy weight will be based on self-report. Height measurement and pre-pregnancy weight will be used to calculate BMI (kg/m^2^), a BMI of 29 or above accounts as inclusion criteria. At each measurement women will be weighed on calibrated electronic scales (SECA 888; SECA 877) wearing no shoes and light clothes, to the nearest 0.1 kg; the average value of two measurements will be used. At baseline and at 35–37 weeks of gestation, relative body fat will be estimated after measurement of subcutaneous skinfolds (biceps, triceps, suprailiac and subscapular) with a Harpenden caliper (British indicators, Sussex, England) according to the method described by Durnin and Womersly (1974) [33]. The measurement is performed twice and the mean value is used. In case the two measurements differ more than 1.0 mm, the skinfold is measured a third time and the mean value of the three values is used. At baseline, waist circumference will be measured twice at the level midway between the lowest ribcage and the iliac crest [34], the average of these two values is taken. When the first measure taken differs from the second measurement by more than 3 cm an additional measure is taken. Neck circumference will be obtained three times during pregnancy in a standing relaxed upright position between mid-cervical spine and mid-anterior neck, to within 1 mm [35].The blood pressure (diastolic and systolic) and heart rate (OMRON 705, Kyoto, Japan), will be recorded on the left arm with an appropriate-sized cuff after the participant had been seated for at least 2–3 minutes. The average value of two measurements taken one minute apart will be used.

##### Fetus/baby

Prenatal examination will be performed by obstetrical ultrasound measurements and in the postnatal period the baby will have a physical examination within 48 hours after birth.

Ultrasound examination of fetal growth and development will be performed during the first trimester at 24–28 and 35–37 weeks of gestation. If the baseline measurement is beyond 16 weeks of gestation, at least one early ultrasound record is mandatory (<14 weeks) with a crown rump length (CRL) [36], to define an accurate date of gestation and hence an estimated date of delivery. The first trimester ultrasound includes the following measurements: biparietal head diameter, head circumference, the abdominal circumference and the femur length to calculate the estimated fetal weight (EFW) [37]. Optional are the CRL [36] and nuchal translucency [38], depending on the gestational age of examination and the fetal liver size measurement [39-41]. Doppler measurements include the Ductus Venosus (DV) (pulsatility index, presence of the a-wave) and the maternal uterine arteries (Uaa) (pulsatility and resistance index) [42]. Also placental location is defined. At 24–28 and 35–37 weeks the fetal biometry will again be defined, except for the CRL, to calculate the EFW. Additionally the definition of the amniotic fluid Index as a marker of fetal well-being and development is defined [43]. Doppler measurements are expanded with the umbilical artery (pulsatility and resistance index), umbilical vein (maximum velocity and presence of single, double or no pulsations) and the middle cerebral artery (pulsatility and resistance index with the maximum peak systolic velocity) additional to the DV and Uaa [42,44-46]. All fetal growth and Doppler measurements have been described intensively for fetal monitoring in obstetrical care and defined by the International Society of Ultrasound in Obstetrics and Gynecology (ISUOG) guidelines [47]. At 24–28 and 35–37 weeks,more innovative measurements concerning fetal body composition evolution are also explored in some participating sites with sufficient expertise. These non-invasive intrauterine measurements consists of fetal fat mass and fetal lean mass, area of the upper arm (midportion of the humerus) and upper leg (midportion of the femur), together with the abdominal fat thickness, subscapular fat thickness [48-53] and the fetal liver size [40,41].

At birth both the placenta and the arterial and venous cord blood will be sampled and processed. The placenta weight (g) will be measured after the removal of the umbilical cord and the membranes using a calibrated scale with minimal precision up to 10 grams.

The newborn baby is examined immediately after birth (one and five minutes) on five criteria on a three point scale (0, 1 or 2), resulting in the Apgar scores with a maximum score of ten [54,55].

Within 48 hours after birth the baby’s weight, length and head circumference will be measured, as well as anthropometry measurements (abdominal circumference, upper- and lower arm and upper- and lower leg circumferences) with skin fold measurements of triceps, quadriceps, subscapular and suprailiac region in mm [52,56-58].

The skinfold measurements are performed with a Harpenden skinfold caliper with a 0.2 mm accurate scale [33]. Every skinfold target is measured at pre-defined spots: triceps skinfold, midway between the acromion and olecranon; the subscapular skinfold, at the lower angle of the scapula; the flank skinfold, in the mid-axillary line just above the crest of the ilium and the thigh skinfold, midway between the crural fold and the large semilunar crease above the patella when the leg is fully extended in the longitudinal plane of the leg. The skinfold will be measured lifting the skin with the thumb and index finger, taking care not to include any underlying tissue. Each measurement will be repeated once and if a difference of more than 0.2 mm is registered, a third measurement is performed and the average of the three is taken.

An additional means of gathering information on neonatal lean and fat mass development is total body fat electrical conductivity (TOBEC). The decision to measure subcutaneous skinfolds instead of TOBEC in the neonate was taken because skinfold examination allows the evaluation of the body fat distribution on trunk and extremities, which may be more helpful in identifying the specific risk feature of T2D of central obesity [53,59].

#### Oral glucose tolerance test (OGTT)

At baseline, 24–28 and 35–37 weeks an OGTT is performed. After an overnight fast of 10 hours, a fasting blood sample is collected, immediately followed by the administration of 250 ml 75 g glucose drink (within a period of 5 minutes). Further blood collections take place at the time points 60 and 120 minutes in which only glucose and insulin will be assessed. At some sites blood will also be collected at 30 and 90 minutes. Participants are instructed not to walk, eat or drink extensively during this test. For clinical practice use and inclusion in the study glucose will be measured in the blood samples at 0, 60 and 120 minutes in the local laboratories at baseline and 24–28 weeks of gestation, some centers will locally analyze the 35–37 weeks sample as well.

#### Biological samples

Blood will be collected from the mother at the time of the OGTT and within 48 hrs after delivery, and from the newborn immediately after delivery (approximately 16 ml of arterial and 16 ml of venous blood from placental chorionic vessels). The samples will be centrifuged and separated aliquots (1000 μl or 250 μl) will be placed in microrack tubes and stored at -20° or -80°C until further analysis in the central trial laboratory in Graz, Austria.

Four placental biopsies, each from the central part of one of the four quadrants in relation to the cord insertion will be taken. These pieces will be equally divided in a maternal and fetal part and stored in a cryotube. The cryotube will be filled with RNA-later (Sigma-Aldrich, St. Louis, MO, USA) and kept at 4°C for at least 24 hours to allow the RNA-later to fully penetrate the tissue. Thereafter the cryotubes will be stored in a freezer of at least −20°C until shipment and further analysis in Graz.

#### Laboratory analyses

From maternal fasting blood samples the concentrations of plasma glucose, 3ß-OH-butyrate, lipids (triglycerides, free fatty acids, total cholesterol, high density lipoprotein cholesterol and low density lipoprotein cholesterol), insulin, leptin, HbA1c, vitamin A, vitamin D and carotenoids will be determined.

From arterial and venous neonatal blood samples, the levels of plasma glucose, 3ß-OH-butyrate, triglycerides, vitamin D, C-reactive protein, leptin and erythropoietin will be measured.

C-reactive protein, insulin, leptin and erythropoietin concentrations will be quantified using commercially available Enzyme-Linked Immuno Sorbent Assay (ELISAs), vitamin D levels by liquid-chromatography coupled with mass spectrometry (LC-MS) and vitamin A and carotenoid levels by high-performance-liquid-chromatography (HPLC) and Ultraviolet (UV) detection. The concentrations of all other analytes will be quantified by conventional clinical chemistry methods.

#### Behaviour

To assess the amount of physical activity the pregnancy physical activity questionnaire (PPAQ) [60] will be used. This questionnaire covers the time spent on light and sedentary behaviour and it is structured into duration per day or week during the current trimester of pregnancy. The PPAQ is validated in pregnancy and reliable to use in European countries [60]. In addition the participants will wear an accelerometer (Actigraph GT3X+, GT1M or Actitrainer), which offers an objective way to assess physical activity [61]. The Actigraph is a tri-axial accelerometer developed and manufactured in Pensacola, Florida, USA. The lightweight device has a sampling frequency of 60–80 Hz. The unit of data derived from the accelerometer is counts/minute. Using the Freedson cut-off points [62] the number of minutes per day in light, moderate and vigorous activity will be calculated as well as time spent sedentary. Participants will wear this device at least for three days, including two days during the week (Monday-Friday) and one day in the weekend (Saturday or Sunday) or a holiday day. Data from waking up until going to bed will be used for analysis. The participants will be asked to remove the accelerometer while swimming, showering or bathing, because it is not waterproof. At such non-wearing intervals participants will be asked to write down what they did in an activity log.

For the purpose of the intervention the dietary behaviour questions will be closely linked to sub-behaviours targeted in the intervention. Therefore a list of 12 items has been formulated on which women can indicate the frequency (days per week) and amount (as large as the size of the palm of a hand or equal to 200 ml for fluids) of the specified food consumed. The list exists of the following items: vegetables, fish, meat/eggs, high fat milk products, fruit, potatoes/pasta, cakes/muffins, whole grain bread, fruit juice, non-diet soft drinks, fast food and breakfast.

To complement this 12-item list is a three-day-food diary, in which participants keep track of what they eat and drink as detailed as possible for three days, including one weekend day. Food and drinks consumed are noted for all meals (breakfast, lunch, dinner) and snacks (in betweens), with quantities, brand names, and preparation methods included where appropriate. Preferably this is done in the same period as wearing the accelerometer. For the purpose of interpretation, the three-day-food diary records will be translated into English and analysed with food nutrition tables and a coding book. Average daily kilojoules (energy intake), percentage total fat, saturated fat, carbohydrate, protein, fibre and the amount of vegetables will be calculated.

Paternal and maternal smoking behaviour will be assessed regarding smoking status, reasons and date of quitting or amount of current cigarette consumption and change in consumption. Alcohol consumption and change in alcohol consumption due to pregnancy will be asked at baseline.

During pregnancy short sleep duration and specially snoring have been associated with abnormal glucose tolerance and hypertension [63-65], so sleeping habit and snoring will be taken into account.

#### Vitamin D

In the questionnaire days travelled to a warmer climate in winter or spring and number of sun beds will be asked. The month of blood collections will be noted to link this to vitamin D content.

The use of multivitamin pills and their dosage will be recorded and the amount of vitamin D/placebo pills missed (compliance) will be noted based on self-report and by pill counter at the measurements session from returned bottles.

Adverse events like hypercalciuria (≥2.27 mmol/mmol calcium/creatinine) and hypercalcaemia will be checked for locally at all measurements (at 24–28 weeks >9.0 mg/dl | 2.25 mmol/l; and at 35–37 weeks > 9.7 mg/dl | 2.43 mmol/l).

#### Psychosocial factors

Based on the Health Action Process Approach (HAPA) model [66] we will assess perceived importance, outcome expectancy, self-efficacy and intention related to weight control, physical activity and nutrition. These factors have been shown to be important predictors in healthy pregnant women or in women with (previous) GDM [67-69] and captured with single validated items that have been used in previous studies. The stage of change questions for physical activity and nutrition behaviour are adapted from Te Wai O Rona: Diabetes Prevention Strategy [19], itself adapted from Prochaska [70]. Mental Health (stress, depression, anxiety) has been shown to affect birth weight and gestational age [71]. Therefore we chose to measure mental health with the widely-used WHO well being index [72] and pregnancy specific worries with the Cambridge worry scale [73]. To measure health related quality of life the EuroQol (EQ 5D) will be used [74].

#### Other

Social demographics, like household composition, marital status, ethnicity (maternal, paternal and of grandparents), education, employment, occupation, shift work, parity, age and paternal information will be obtained.

In the questionnaire information on pre-existing conditions is included: family history of diabetes, previous GDM diagnosis, IGT, hypertension and PCOS. Past/current obstetric history and medication / vitamin use is assessed also.

Co-morbidities, sick leave and complications during pregnancy or delivery will be assessed. Obstetrical and perinatal complications will be registered, such as spontaneous or induced abortion before 22 weeks, intra-uterine death at or after 22 weeks gestation, hypertensive diseases, preeclampsia, pre-term delivery (<37 weeks), need for labour induction or augmentation, assisted vaginal delivery, shoulder dystocia, maternal haemorrhage and caesarean section. Also adverse events for the neonate, such as hypoglycaemia, hyperbilirubinaemia (with or without therapy), respiratory distress, congenital malformation, admission to the neonatal (intensive) care unit, stillbirth, early or late neonatal death will be registered.

### Primary outcome measures

For assessing intervention effects, the primary outcomes are maternal weight gain, fasting glucose levels and insulin sensitivity. Maternal weight gain will be defined as the weight change from baseline measurement to the last measurement at 35–37 weeks of gestation. Insulin sensitivity will be derived from homeostasis model assessment (HOMA) [75].

#### Economic evaluation

This study will include an economic evaluation to assess the cost-effectiveness of the intervention.

All direct costs, medical and nonmedical, and indirect cost, morbidity and mortality, will be registered.

Regarding economic evaluation of prevention strategies, the cost-effectiveness analysis will be carried out from a societal perspective. Moreover, we will assume the following general principles when performing the analysis: 1) intention to treat 2) the time horizon will be extended to 10 weeks postpartum to register all cost and benefits, and 3) an assessment of uncertainty in final outputs will be applied. In this case, univariate and multivariate analyses of strategies and scenarios (including discounting rates) will be undertaken. Finally a multivariate analysis of possible scenarios related to epidemiological variations, costs and benefits will be performed. Furthermore, cost-effectiveness, and cost utility mean ratios of each intervention will be calculated and compared. Incremental cost-effectiveness ratios (Δ Costs /Δ Effectiveness) and net health benefits (according to willingness to pay) of each intervention will be calculated. Additionally, the degrees of dominance of competing strategies will be analyzed by using the efficiency frontier graphs. Univariate (ie. tornado graphs- for detecting the most sensitive parameters) and multivariate sensitivity analyses (ie. confidential intervals and nonparametric-bootstrapping) will be performed. Moreover, a budgeting impact of the introduction of the most efficient strategies will be carried out aiming to highlight the feasibility of introducing those strategies. Finally, data from this prospective study will enable us to perform a decision modeling for health economic evaluation of the introduction of the different strategies in Europe to prevent GDM in time horizons of 3 and 5 years and applying a discounting rate for costs and benefits (ie. 3.5%). This model will allow investigators to estimate cost-effectiveness of the interventions in simulated cohort studies in each of the sites.

### Database

All trial subject data is entered into a web-based electronic database using the Microsoft.Net development environment that is accessible from any web-enabled computer, tablet or cell phone. The database is housed on an SQL server that employs a certificate and is housed in a physically-secure location. Pages are in English and employ drop-down menus, pick-lists and text boxes, and are encrypted with Standard SSL level encryption. Operator entry is password protected. No personal identifiers for study subjects are housed within the database; rather they are cross-referenced to a unique study number, the first two digits of which indicate the geographic clinical site. The security and confidentiality standards of the database are compliant with those required under HIPAA privacy rules established by the National Institutes of Health, U.S.A., and the recommendations of EuroSOCAP. Back-up copies of the entire database are made daily. A Data Management Board has been created to oversee the integrity of data and to determine its academic usage.

### Process evaluation

Process evaluation [76] will be used to gain insight into the response rate of the target population (reach), behaviour changes, implementation, fidelity (the extent to which the intervention was delivered as planned), delivered dose (the amount of the intervention actually delivered) and participants perceptions (satisfaction). For this the recruitment database, counselling logs on the PDA, evaluation questionnaire and the MITI 3.1.1 code [25] will be used.

### Data analysis

Multilevel analyses will be performed, to take the clustering effect into account, since the success of treatment can be dependent on the skills of the provider as well as specific characteristics of a treatment centre. Longitudinal, linear regression analyses will be performed to assess intervention effects. In these analyses, the correlation between multiple measurements within one individual is taken into account. The regression coefficient reflects the average difference in the outcome variable between conditions. Possible confounders will be entered into the regression analyses. Confounding will be defined as a change in the regression coefficient of 10% or more. Possible effect modification will be studied as well, defined as a significant (p <0.10) interaction-term between group allocation and the variable concerned. Data will be analysed according to the intention-to-treat principle.

Interactions between interventions will be assessed, and when not present, groups will be combined.

## Discussion

Our study is unique in that it is conducted Europe-wide and aims to develop preventive measures to lower the incidence of GDM. The combination of preventing excessive gestational weight gain, by improving physical activity and healthy eating alongside vitamin D supplementation is thought to improve insulin sensitivity and prevent an increase in blood glucose levels. This should ultimately reduce the risk of macrosomia in the infant and future overweight/obesity and T2D.

The goal to limit gestational weight gain to a maximum of 5 kg, is below the current cut off points for the obese category [21]. However, these gestational weight gain cut off points were defined using limited data available at that time. Recent research advocates for less gestational weight gain, at least for severe obese women, since it is associated with less macrosomia or large for gestational age infants [77-79]. Insight into the diet and physical activity patterns that lead to less weight gain or even weight loss can be helpful to these recommendations and might explain some of the negative effects associated with excessive weight loss, such as small for gestational age babies.

MI [80] and goal setting [22] are promising strategies in helping behaviour-related changes to occur. While evidence on lifestyle interventions in overweight pregnant women is still scarce [11], and present with unique challenges, pregnancy offers an excellent window of opportunity for lifestyle change, as women are motivated by the will to care for their growing infant. MI is suitable to assist the women in reviewing their ambivalence in a non-confronting way and help translate intrinsic motivation into actual behaviour changes. It is anticipated that barriers to change may shift throughout the pregnancy period, demanding continued tailored support to keep women on track with managing their gestational weight gain, healthy eating and physical activity. Combining simple understandable and relevant health messages with a detailed manual and a lifestyle coach is expected to be sufficiently effective to attain the desired weight management goal.

An effect of vitamin D on several pregnancy outcomes is suggested [81]. However, whether the same association between a deficiency of maternal vitamin D and the incidence of GDM exist in randomised control trials as in observational studies has still to be established [82]. The DALI study will aim to close this gap in knowledge and will evaluate the potential effectiveness of vitamin D supplementation on the risk of developing GDM, something which is needed especially for certain population groups, but also for the countries and the rising incidence of GDM and T2D [83]. DALI will also be the first study to assess whether vitamin D supplementation will provide additional benefits on the capability to reduce the risk of GDM beyond lifestyle alone.

Biobanking on a European level has been recognised as one of the major resources promoting biomedical research. This will allow future comparison of the metabolic phenotype and genotype of responders and non-responders after consideration of the psychological and situational characteristics of participating women. The robust trial monitoring and methods to maintain fidelity of the trial will maximise the confidence that any difference in response is due to differences between participants rather than differences in the way the trial was delivered. Maintaining fidelity is something of particular importance when intervening across 9 countries with their own languages, cultures and health systems.

The information obtained from this trial can be used to inform and develop public health policies Europe-wide in pregnancy and pregnancy related prevention of GDM. The overall aim of the DALI study is to identify the best available measures to prevent GDM in an ongoing pregnancy, to provide cost-benefit calculation of GDM prevention for health care systems, and to establish a pan-European cohort of mother-offspring pairs for future analyses with a central biobank and database.

## Abbreviations

ACOG: American college of obstetrics and gynaecology; BMI: Body mass index; CRL: Crown rump length; DV: Ductus venosus; EFW: Estimated fetal weight; ELISAs: Enzyme-linked immuno sorbent assay; EQ 5D: EuroQol; F2F: Face to face; GDM: Gestational diabetes mellitus; HAPA: Health action process approach; HE: Healthy eating; HOMA: Homeostatis model assessment; HPLC: High-performance-liquid-chromatography; IGT: Impaired glucose tolerance; IADPSG: International association of diabetes and pregnancy study group; IOM: Institute of medicine; ISUOG: International society of ultrasound in obstetrics and gynecology; LC-MS: Liquid-chromatography coupled with mass spectrometry; MI: Motivational Interviewing; MITI: Motivational interviewing treatment integrity; NICE: National institute for health and clinical excellence; OGTT: Oral glucose tolerance test; PA: Physical activity; PCOS: Polycystic ovarian syndrome; PDA: Personal digital assistance; PPAQ: Pregnancy physical activity questionnaire; TOBEC: Total body fat electrical conductivity; T2D: Type 2 diabetes; Uaa: Uterine arteries; UV: Ultraviolet; WHO: World health organisation; 25OHD: 25-hydroxyvitamin D.

## Competing interests

The authors declare that they have no competing interests.

## Authors’ contributions

All authors contributed to the conception and design of the trial, read and corrected draft versions of the manuscript and approved the final manuscript.

## Pre-publication history

The pre-publication history for this paper can be accessed here:

http://www.biomedcentral.com/1471-2393/13/142/prepub
